# Effects of Computer Navigation versus Conventional Total Knee Arthroplasty on Endothelial Damage Marker Levels: A Prospective Comparative Study

**DOI:** 10.1371/journal.pone.0126663

**Published:** 2015-05-08

**Authors:** Shu-Jui Kuo, Feng-Sheng Wang, Ching-Jen Wang, Jih-Yang Ko, Sung-Hsiung Chen, Ka-Kit Siu

**Affiliations:** 1 Department of Orthopedic Surgery, China Medical University Hospital, Taichung, Taiwan; 2 Department of Medical Research, Chang Gung Memorial Hospital—Kaohsiung Medical Center, Kaohsiung, Taiwan; 3 Department of Orthopedic Surgery, Chang Gung Memorial Hospital—Kaohsiung Medical Center, Kaohsiung, Taiwan; Cardiff University, UNITED KINGDOM

## Abstract

Total knee arthroplasty (TKA) inevitably perturbs the femoral medullary canal, which increases blood loss or morbidities associated with marrow embolization postoperatively. Computer navigation TKA reportedly minimizes medullary disturbance to alleviate perioperative blood loss. We performed a prospective comparative study, enrolling 87 patients with osteoarthritic knees from March 2011 to December 2011 in our hospital. The patients were separated into two groups, according to the surgeon they visited. Fifty-four patients underwent computer navigation TKAs and 33 had conventional TKAs. Levels of cell adhesion molecules (CAMs), including intercellular adhesion molecule-1 (ICAM-1), vascular cell adhesion molecule-1 (VCAM-1), and platelet endothelial cellular adhesion molecule-1 (PECAM-1) in sera and hemovac drainage were measured by ELISA before and 24 hours after the surgery. We showed that patients receiving computer navigation TKAs had less blood loss and lower CAMs in serum and hemovac drainage after the operation. Less postoperative elevation of serum ICAM-1 (p=0.022) and PECAM-1 (p=0.003) from the preoperative baseline after the surgery was also noted. This study provides molecular evidence for the differential extent in vascular injury between conventional and navigation TKAs and sheds light on the possible benefits of computer navigation TKAs.

**Trial Registration:**

ClinicalTrials.gov NCT02206321

## Introduction

Total knee arthroplasty (TKA) is a well-established modality with a high satisfaction rate for various knee disorders[1]. However, this surgical procedure inevitably perturbs the femoral medullary canal, leading to marrow embolization that reportedly increases the risk of myocardial infarction or cardiac stress postoperatively[2, 3]. Minimizing femoral medullary canal destruction, thus reducing marrow embolism-related morbidities, is an important issue for TKA surgeries.

In addition to improving prosthetic alignment, computer-assisted navigation TKAs also contribute to reduced operative blood loss and systemic emboli[4–8]. These observations imply that navigation TKAs may cause less microvascular damage than conventional TKAs. However, the molecular evidence for the differential extent in vascular injury between conventional and navigation TKAs remains elusive.

Intercellular adhesion molecule-1 (ICAM-1), vascular cell adhesion molecule-1 (VCAM-1), and platelet endothelial cellular adhesion molecule-1 (PECAM-1) are cell adhesion molecules (CAMs) that contribute to endothelial activation and leukocyte recruitment[9]. They have been employed as markers for endothelial or vascular damage or hemorrhage, including coronary artery disease[10–12]. After total joint surgeries, patients reportedly had higher serum levels of leucocytes and endothelial markers [13]. Therefore, we postulated that serum levels of CAMs in patients receiving navigation TKAs may be different from those receiving conventional TKAs.

The purpose of this prospective comparative study was to compare ICAM-1, VCAM-1 and PECAM-1 levels in serum and hemovac drainage of patients receiving navigation and conventional TKAs. We hypothesized that navigation TKAs would lead to less postoperative elevation of serum CAMs compared to preoperative baselines than conventional TKAs.

## Materials and Methods

### Patients

The protocol for this trial and supporting TREND checklist are available as supporting information (see S1 TREND Checklist and S1 Protocol).

This prospective comparative study was approved by the Institutional Review Board of Kaohsiung Chang Gung Memorial Hospital (IRB 100-0038A3) before recruiting the patients, and was conducted from March 2011 to December 2011 (see S1 IRB). Local officials did not mandate registration before recruiting the patients, but this study was later registered in the ClinicalTrials.gov system (ID: NCT02206321). Our study adhered to the TREND reporting statements.

Patients in need of TKA surgery due to degenerative osteoarthritis of the knee visited the outpatient department first and then were scheduled for admission for TKA surgery. They were self-separated into two groups when they visited the outpatient department. Those patients who visited Dr. CJ Wang would undergo conventional TKA, and those who visited Dr. JY Ko would undergo computer navigation TKA. Both senior surgeons had performed more than 1,000 TKAs using the conventional and the computer navigation method, respectively, before the beginning of our study. The patients did not know which surgeon performed conventional or computer navigation TKA before admission. After admission and before the surgery, all of the patients were asked whether they consented to participate in this study. All but three patients consented to participate. The patients knew their allocation as they completed written informed consent, because there were four additional small skin incisions (0.5 cm each) for the insertion of reference arrays for the navigation procedures. No patients shifted to the other group during the study. Those with autoimmune diseases, rheumatoid arthritis, malignancies, previous knee surgery or post-traumatic arthritis were excluded.

All surgical procedures, including aseptic dragging and skin preparation were performed with standard protocols in a standard surgical facility. Each patient was given intravenously a single dose of 1 mg prophylactic cefazolin and a pneumatic tourniquet (300 mm Hg), followed by a mid-vastus approach after midline skin incision.

Because of the pilot nature of the study, we planned to enroll at least 30 patients for each group after consultation with the statistician.

### Navigation-assisted TKA

The bone cuts were extramedullary-guided and mapped using navigation and infrared-based systems (Vector Vision; Brain LAB, Heimstetten, Germany), according to the manufacturer’s instructions. Briefly, two fixed reference arrays with marker spheres were tracked using an infra-red camera, and the marker spheres were fixed to the distal femur (4 mm pins) and proximal tibia (3 mm pins) via two bi-cortical half pins. The hip joint center, distal femur and proximal tibia articulating surfaces, and the medial/lateral malleolus of the ankle were mapped and registered. The femoral component was referenced to the anterior cortex of the distal femur. A multiple-referencing method using epicondylar line, Whiteside line, and posterior condylar line was adopted to determine the appropriate rotation of the femoral component, and the size and position of the femoral prosthesis was optimized by the navigation system. The distal femur cut and chamber cut were guided by the real-time navigation system without reaming and destroying the bone marrow cavity.

An extramedullary guide connected to a reference array was employed to determine the tibia cutting level, varus-valgus angle and tibia slope. The rotation of the tibia component was adjusted to fit the femoral component. An extramedullary guiding rod was used to reference the center of the anterior ankle joint, assisting the determination of the tibial component rotation. Bone cut was achieved under real-time navigation.

After completing the femur and tibia bone cut, femoral and tibia components (LPS-Flex system, Nexgen; Zimmer, Warsaw, IN, USA) were implanted with antibiotic-loaded cement fixation. A neutral mechanical axis with a deviation of less than 1 degree was obtained after soft tissue balancing, with the assistance of a real-time computer screen. A 1/8-inch hemovac (Zimmer Haemovac; Zimmer, Warsaw, IN, USA) was inserted as a closed drainage system, and then removed 24 hours after surgery.

### Conventional TKA

Femoral bone cuts (distal and chamber cuts) were guided by an intramedullary system and guiding instruments. The proper size and positioning of the prosthesis (LPS-Flex system, Nexgen; Zimmer, Warsaw, IN, USA) was based on the surgeon’s experience, and was followed by the implantation of the prosthesis with antibiotic-loaded cement fixation. A 1/8-inch hemovac (Zimmer Haemovac; Zimmer, Warsaw, IN, USA) was inserted as a closed drainage system and removed 24 hours postoperatively.

### Perioperative management

Administration of anti-coagulants was halted 1 week before surgery and resumed on the day after surgery. Patients without previous use of anti-coagulants received aspirin for chemical prophylaxis if no contraindications. Intravenous cefazolin 1 g every 8 hours was administered for 3 doses after surgery. The hemovac drainage was kept in place for 24 hours, and then removed. Hemoglobin and hematocrit levels were measured before surgery and 24 hours after the operation. If patients had hemoglobin < 8 mg/dL or hemoglobin 8–9 mg/dL with unstable vital signs, blood transfusion with packed red blood cells was provided. After removing the hemovac drainage, patients proceeded with continuous passive motion, including active flexion-extension, quadriceps training and walker-aided ambulation with the assistance of physical therapists. Patients with a clean operative wound and stable general condition were discharged if range of motion of the knee exceeded 95 degrees. All complications before manuscript preparation (July 2014) were scrutinized and reported.

### ELISA assessment

Five milliliters of drainage from the hemovac was harvested and processed to collect supernatants. Ten milliliters of peripheral blood was drawn from each patient before and 24 hours after TKA and processed to collect sera. All processed supernatants and sera were stored at -80°C till ELISA analysis. Concentrations of all CAMs in sera and drainage supernatants were measured by respective ELISA kits (R & D Systems), according to the manufacturer’s instructions. The technician performing the ELISA analysis was blinded to the patient profile and grouping via the de-labeling process.

### Blood loss assessment

Blood loss during the first 24 hours was calculated by the method proposed by Sehat et al. and other authors [14, 15], which can be summarized by the following formula: blood loss = total blood volume × (Hct_pre-op_-Hct_post-op_)/ Hct_mean_ + volume transfused

where total blood volume was calculated based on sex, height and weight, using the method described by Nadler et al[16] (https://www.easycalculation.com/medical/blood-volume.php).

### Statistical analyses

Data are expressed as median (lower quartiles, upper quartiles). Categorical variables were compared by Chi-square test. As for continuous variables, the Mann-Whitney U test and Wilcoxon signed-rank test were utilized for between-group and within-group comparisons, respectively. The Spearman’s correlation coefficient was used to measure the strength of correlation between the two variables. All statistics were performed by SPSS software, and a p value < 0.05 was regarded as statistically significant.

## Results

**Fig 1:**
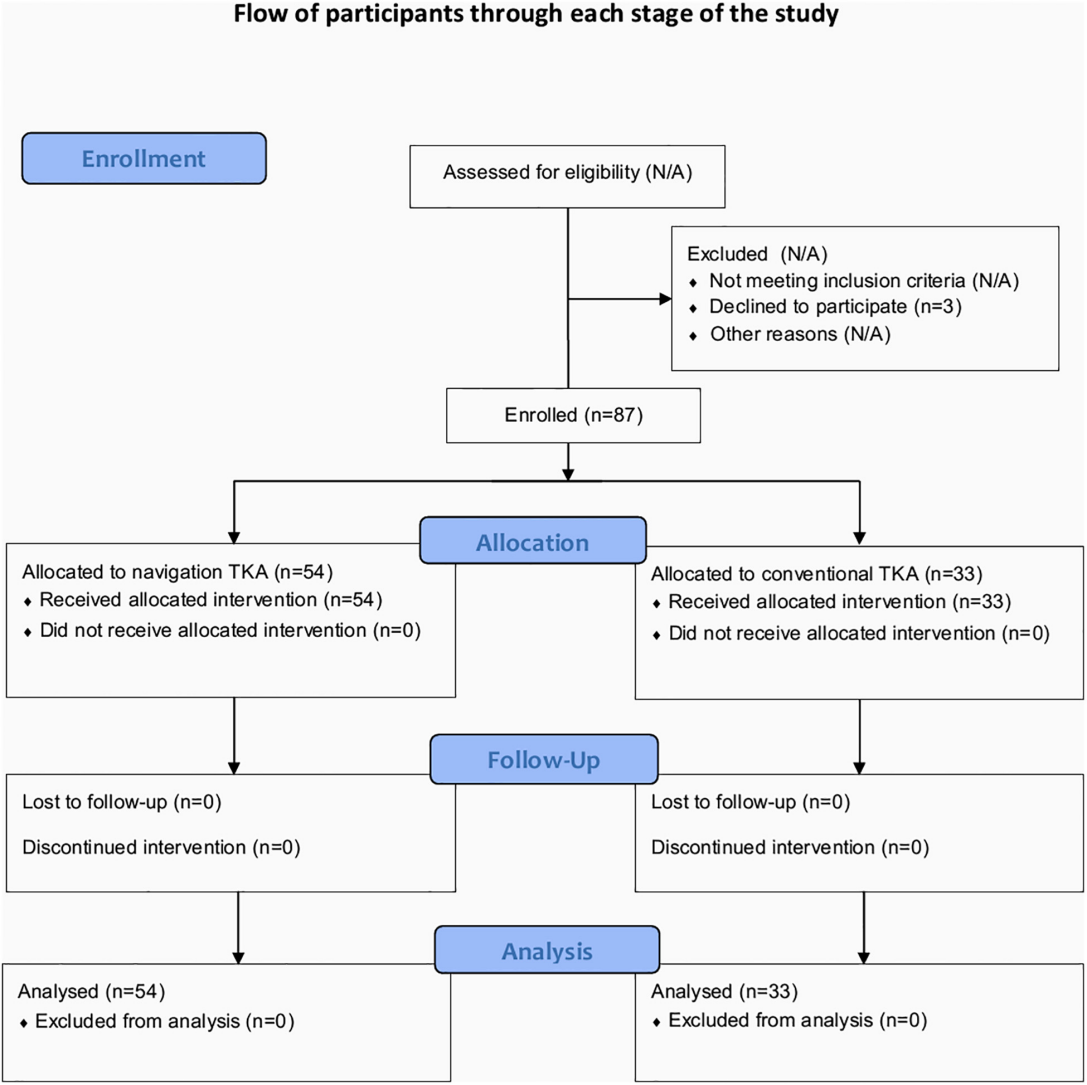
Flowchart of the participants through each stage of the study.

Ninety patients met the inclusion criteria from March 2011 to December 2011. Three of the 90 patients that underwent TKA surgery declined to participate and were excluded from the study. Finally, 87 eligible patients provided written informed consent and were enrolled. Fifty-four patients underwent computer-assisted TKAs and 33 received conventional TKAs. All 87 patients completed the preoperative and postoperative blood sampling and the collection of drainage from the hemovac (Fig 1).

There were no significant differences in gender, affected side, age or BMI between the computer navigation and conventional TKA group. The numbers of patients afflicted with coronary artery disease and coronary artery equivalent disease (including symptomatic carotid artery disease, peripheral arterial disease, abdominal aortic aneurysm, diabetes, and chronic kidney disease) in both groups were not significantly different (Table 1)[17].

**Table 1:** Baseline clinical and radiographic characteristics of patients undergoing conventional or navigation TKA surgery.

	Conventional	Navigation	P value
Gender (Male/Female)	10/23	14/40	0.66
Side (L/R)	17/16	28/26	0.98
Age (years)	71.0 (67.0, 78.0)	71.0 (66.0, 74.0)	0.35
BMI	26.2 (24.6, 29.8)	28.6 (25.7, 30.2)	0.16
CHD / equivalent	11/22	16/38	0.72

The calculated volume of blood loss in the computer navigation group was 955 (772, 1164) mL, significantly lower (p = 0.001) than the 1265 (963, 1475) mL in the conventional group.

The skin to skin closure time was 116 (103, 124) minutes in the navigation group and 110 (101. 115) minutes in the conventional group. (p = 0.141)

The baseline serum CAMs did not differ between the two groups before surgery. Of interest, postoperative serum ICAM-1 (Table 2), VCAM-1 (Table 3) and PECAM-1 (Table 4) levels in the computer navigation group were 35.5% (p <0.001), 2.0% (p = 0.037) and 49.3% (p <0.001) lower, respectively, than those in the conventional group. The extent of postoperative elevation of serum ICAM-1 (p = 0.022) and PECAM-1 (p = 0.003) in the navigation group was significantly milder than that in the conventional group. However, there was no significant difference (p = 0.435) in the postoperative elevation of VCAM-1 between groups (Tables 2–4).

**Table 2:** Serum concentrations of ICAM-1(unit: ng/mL) in patients before and after navigation and conventional TKAs, and the changes from preoperative baseline after surgery (post-OP—pre-OP).

ICAM-1	Before operation	After operation	Changes
Navigation	155.3 (140.7, 170.4)	158.1 (138.9,183.8)	-1.0 (-5.8, 11.5)
Conventional	168.9 (132.6, 196.4)	245.9 (206.7,274.6)	1.0 (-1.3, 19.4)
p-value	0.28	< 0.001	0.022

**Table 3:** Serum concentrations of VCAM-1 (unit:ng/mL) in patients before and after navigation and conventional TKAs, and the changes from preoperative baseline after surgery (post-OP—pre-OP).

VCAM-1	Before operation	After operation	Changes
Navigation	489.2(466.1,517.4)	580.0 (566.3,590.5)	105.1 (81.2, 118.9)
Conventional	495.6(449.9,557.6)	592.6 (532.3,642.2)	103.1 (85.5, 122.3)
p-value	0.656	0.037	0.435

**Table 4:** Serum concentrations of PECAM-1 (unit: pg/mL) in patients before and after navigation and conventional TKAs, and the changes from preoperative baseline after surgery (post-OP—pre-OP).

PECAM-1	Before operation	After operation	Changes
Navigation	1071.1(570.6,1853.4)	2243.4 (717.8,3716.6)	1121.1 (212.2, 3213.1)
Conventional	1498.9(636.9,4378.9)	4429.4 (3460.0,6286.7)	2899.1 (1901,7, 3558.3)
p-value	0.152	<0.001	0.003

The patients in the computer navigation group had lower ICAM-1 levels in hemovac drainage supernatants than patients in the conventional group. The ICAM-1 (Table 5) and VCAM-1 (Table 6) levels in hemovac drainage supernatants were significantly correlated with those in sera. However, PECAM-1 levels in hemovac were not significantly correlated with those in sera (Table 7).

**Table 5:** Correlation of ICAM-1 concentrations (unit: ng/mL) in hemovac samples and postoperative serum samples.

ICAM-1	Hemovac	Post-OP serum	Correlation coefficient
Navigation	123.1 (103.9,151.9)	158.1 (138.9,183.8)	0.62 (p<0.001)
Conventional	148.0 (120.2,167.8)	245.9 (206.7,274.6)	0.54 (p = 0.007)
p-value	0.005	<0.001	

**Table 6:** Correlation of VCAM-1 concentrations (unit: ng/mL) in hemovac samples and postoperative serum samples.

VCAM-1	Hemovac	Post-OP serum	Correlation coefficient
Navigation	437.4 (356.8,534.2)	580.0 (566.3,590.5)	0.61 (p<0.001)
Conventional	481.2 (348.1,616.2)	592.6 (532.3,642.2)	0.70 (p<0.001)
p-value	0.280	0.037	

**Table 7:** Correlation of PECAM-1 concentrations (unit: ng/mL) in hemovac samples and postoperative serum samples.

PECAM-1	Hemovac	Post-OP serum	Correlation coefficient
Navigation	9004.2 (7009.7,12542.3)	2243.4 (717.8,3716.6)	0.15 (p = 0.384)
Conventional	10225.6 (7539.7,12139.3)	4429.4 (3460.0,6286.7)	0.08 (p = 0.692)
p-value	0.300	<0.001	

Up to the time of manuscript preparation (July 2014), two patients, an 85-year-old female in the conventional group and a 64-year old male in the computer navigation group, were admitted for angina symptoms, with an interval between surgery and admission of 12 and 14 months, respectively. Coronary angiography revealed no apparent stenosis for the 85-year-old female patient and single-vessel disease for the 64-year-old male patient. Percutaneous angioplasty was performed smoothly for the 64-year-old male. However, no major complications were noted in any of the patients of both groups postoperatively.

## Discussion

Complications secondary to bone marrow violation are significant concerns after TKA surgeries. Computer navigation TKAs do not involve the reaming of the medullary canal, thus minimizing the destruction of the femoral medullary cavity. While this modality reportedly improves blood loss and prosthesis accuracy, biochemical verification of vascular injury has not been defined[7, 8]. In this study, we provided novel evidence that patients had decreased blood loss concomitant with mitigated postoperative elevation of levels of CAMs after computer navigation TKA, which is indicative of its less-invasive nature with regard to the integrity of the femoral medullary cavity. The results of the current study shed new light on the known advantages of computer-aided TKA procedures for patients.

Compared to patients with conventional TKAs, patients that underwent computer navigation TKAs had milder postoperative elevation of serum ICAM-1 and PECAM-1 from the preoperative baseline. These molecules are associated with vessel damage, thrombosis and hemorrhage, and reportedly promote atherogenesis of various tissues in pathologic contexts[9, 12, 18–21]. In the current study, the milder elevation of postoperative serum CAMs from the preoperative baseline reflects the milder vessel-deleterious reactions after computer navigation TKAs.

Marrow embolism after conventional TKA might be associated with increased risks of acute cardiac disorders[2]. A retrospective study argued that the increased risk of acute myocardial infarction was attributable to the destruction of the femoral medullary canal in the TKA procedure[2]. Serum ICAM-1 levels are linked to macrovascular disease, and serum VCAM-1 concentrations have been correlated with the occurrence of acute coronary syndrome [11, 22, 23]. Although the current study does not correlate the milder elevation of postoperative serum CAMs from baseline to the lower risk of cardiovascular complications in patients after computer-aided TKA, the timing of our observation overlapped exactly with the “hazardous first two weeks” for myocardial infarction [2]. The significance of differential serum CAMs and the correlation with the incidence of heart disorders after different TKA modalities merits long-term follow-up.

We acknowledge the limitation of this study, which is that all participants came from the outpatient department and chose surgeons to perform elective TKA surgery of their own free will. However, the patients did not know which surgeon performed navigation or conventional TKA until the day before the operation, and no patient shifted to the other group during the study. The two physicians were arthroplasty specialists who had performed more than 1,000 TKAs using the method they were familiar with before the study, and neither of them shifted to performing the other technique throughout the course. The shift to a less familiar technique might introduce performance bias, and the choice of a less familiar method would probably prolong the operation time and confound the results. While the current study may not be a double-blinded or truly randomized study design, patient attributes, including gender, age, side in need of TKA, BMI, and heart comorbidities were not significantly different between the groups. The skin to skin closure time for the two groups was similar. All procedures for ELISA analysis were performed using stringent de-coding procedures, and technicians were all blinded to the identity and source of the specimens. Despite all endeavors to minimize possible bias, more rigorously-designed studies, such as randomized double-blind studies, are still necessary to substantiate the preliminary finding. The results of this study cannot be extrapolated to conclude that navigation TKAs lead to fewer postoperative cardiac events and fewer postoperative thromboembolic complications. The correlations between the postoperative CAMs and the incidence of cardiac events or thromboembolic complications warrant a larger study population for further validation.

Taken together, our results reveal that conventional TKAs inevitably perturb the femoral medullary canal, leading to the destruction of vascular integrity, and thereby contribute to more apparent elevations of concentrations of CAMs after the surgery. Computer navigation TKA impedes the medullary canal to a lesser extent, which minimizes vessel deterioration and leads to milder elevation of CAMs after the surgery. This study highlights low serum CAMs as emerging biochemical indicators that strengthen the advantage of navigation TKA. The correlation between differential CAM levels and the incidence of heart events deserves further investigation.

## Supporting Information

S1 IRB The IRB approval document of the local IRB committee. (PDF)

S1_Protocol The protocol of the current study. (DOC)

S1_TREND_Checklist The TREND checklist of the current study. (PDF)

## References

[1] Carr AJ, Robertsson O, Graves S, Price AJ, Arden NK, Judge A, et al Knee replacement. Lancet. 2012;379(9823):1331–40. Epub 2012/03/09. 10.1016/S0140-6736(11)60752-6 . 22398175

[2] Lalmohamed A, Vestergaard P, Klop C, Grove EL, de Boer A, Leufkens HG, et al Timing of acute myocardial infarction in patients undergoing total hip or knee replacement: a nationwide cohort study. Archives of internal medicine. 2012;172(16):1229–35. Epub 2012/07/25. 10.1001/archinternmed.2012.2713 . 22826107

[3] Gandhi R, Petruccelli D, Devereaux PJ, Adili A, Hubmann M, de Beer J. Incidence and timing of myocardial infarction after total joint arthroplasty. The Journal of arthroplasty. 2006;21(6):874–7. Epub 2006/09/05. 10.1016/j.arth.2005.10.007 . 16950042

[4] Hetaimish BM, Khan MM, Simunovic N, Al-Harbi HH, Bhandari M, Zalzal PK. Meta-analysis of navigation vs conventional total knee arthroplasty. The Journal of arthroplasty. 2012;27(6):1177–82. Epub 2012/02/16. 10.1016/j.arth.2011.12.028 . 22333865

[5] Schnurr C, Csecsei G, Eysel P, Konig DP. The effect of computer navigation on blood loss and transfusion rate in TKA. Orthopedics. 2010;33(7):474 Epub 2010/07/09. 10.3928/01477447-20100526-08 . 20608630

[6] Kalairajah Y, Cossey AJ, Verrall GM, Ludbrook G, Spriggins AJ. Are systemic emboli reduced in computer-assisted knee surgery?: A prospective, randomised, clinical trial. The Journal of bone and joint surgery British volume. 2006;88(2):198–202. Epub 2006/01/26. 10.1302/0301-620X.88B2.16906 . 16434523

[7] Kalairajah Y, Simpson D, Cossey AJ, Verrall GM, Spriggins AJ. Blood loss after total knee replacement: effects of computer-assisted surgery. The Journal of bone and joint surgery British volume. 2005;87(11):1480–2. Epub 2005/11/02. 10.1302/0301-620X.87B11.16474 . 16260662

[8] Huang TW, Hsu WH, Peng KT, Hsu RW, Weng YJ, Shen WJ. Total knee arthroplasty with use of computer-assisted navigation compared with conventional guiding systems in the same patient: radiographic results in Asian patients. The Journal of bone and joint surgery American volume. 2011;93(13):1197–202. Epub 2011/07/22. 10.2106/JBJS.J.00325 . 21776572

[9] Galkina E, Ley K. Vascular adhesion molecules in atherosclerosis. Arteriosclerosis, thrombosis, and vascular biology. 2007;27(11):2292–301. Epub 2007/08/04. 10.1161/ATVBAHA.107.149179 . 17673705

[10] Schett G, Kiechl S, Bonora E, Zwerina J, Mayr A, Axmann R, et al Vascular cell adhesion molecule 1 as a predictor of severe osteoarthritis of the hip and knee joints. Arthritis and rheumatism. 2009;60(8):2381–9. Epub 2009/08/01. 10.1002/art.24757 . 19644856

[11] Jude EB, Douglas JT, Anderson SG, Young MJ, Boulton AJ. Circulating cellular adhesion molecules ICAM-1, VCAM-1, P- and E-selectin in the prediction of cardiovascular disease in diabetes mellitus. European journal of internal medicine. 2002;13(3):185–9. Epub 2002/05/22. . 12020626 10.1016/s0953-6205(02)00014-6

[12] Rothoerl RD, Schebesch KM, Kubitza M, Woertgen C, Brawanski A, Pina AL. ICAM-1 and VCAM-1 expression following aneurysmal subarachnoid hemorrhage and their possible role in the pathophysiology of subsequent ischemic deficits. Cerebrovascular diseases. 2006;22(2–3):143–9. 10.1159/000093243 . 16691023

[13] Hughes SF, Hendricks BD, Edwards DR, Maclean KM, Bastawrous SS, Middleton JF. Total hip and knee replacement surgery results in changes in leukocyte and endothelial markers. J Inflamm (Lond). 2010;7:2 Epub 2010/02/12. 10.1186/1476-9255-7-2 20148137 PMC2820000

[14] Sehat KR, Evans RL, Newman JH. Hidden blood loss following hip and knee arthroplasty. Correct management of blood loss should take hidden loss into account. The Journal of bone and joint surgery British volume. 2004;86(4):561–5. . 15174554

[15] McConnell JS, Shewale S, Munro NA, Shah K, Deakin AH, Kinninmonth AW. Reducing blood loss in primary knee arthroplasty: a prospective randomised controlled trial of tranexamic acid and fibrin spray. The Knee. 2012;19(4):295–8. 10.1016/j.knee.2011.06.004 . 21733697

[16] Nadler SB, Hidalgo JH, Bloch T. Prediction of blood volume in normal human adults. Surgery. 1962;51(2):224–32. . 21936146

[17] Linton MF, Fazio S. A practical approach to risk assessment to prevent coronary artery disease and its complications. The American journal of cardiology. 2003;92(1A):19i–26i. Epub 2003/07/18. . 12867251 10.1016/s0002-9149(03)00505-8

[18] Shenkar R, Cohen AJ, Vestweber D, Miller YE, Tuder R, Abraham E. Hemorrhage and resuscitation alter the expression of ICAM-1 and P-selectin in mice. Journal of inflammation. 1995;45(4):248–59. . 8867669

[19] Isogai N, Tanaka H, Asamura S. Thrombosis and altered expression of intercellular adhesion molecule-1 (ICAM-1) after avulsion injury in rat vessels. Journal of hand surgery. 2004;29(3):230–4. 10.1016/j.jhsb.2004.03.001 . 15142692

[20] Polin RS, Bavbek M, Shaffrey ME, Billups K, Bogaev CA, Kassell NF, et al Detection of soluble E-selectin, ICAM-1, VCAM-1, and L-selectin in the cerebrospinal fluid of patients after subarachnoid hemorrhage. Journal of neurosurgery. 1998;89(4):559–67. 10.3171/jns.1998.89.4.0559 . 9761049

[21] Frijns CJ, Kappelle LJ. Inflammatory cell adhesion molecules in ischemic cerebrovascular disease. Stroke; a journal of cerebral circulation. 2002;33(8):2115–22. . 12154274 10.1161/01.str.0000021902.33129.69

[22] Zamani P, Schwartz GG, Olsson AG, Rifai N, Bao W, Libby P, et al Inflammatory biomarkers, death, and recurrent nonfatal coronary events after an acute coronary syndrome in the MIRACL study. Journal of the American Heart Association. 2013;2(1):e003103 Epub 2013/03/26. 10.1161/JAHA.112.003103 23525424 PMC3603244

[23] Mashru MR, Shah VK, Soneji SL, Loya YS, Vasvani JB, Payannavar S, et al Soluble levels of cell adhesion molecules (CAMs) in coronary artery disease. Indian heart journal. 2010;62(1):57–63. Epub 2010/12/25. . 21180036

