# Supplementary material for: Effects of Computer Navigation versus Conventional Total Knee Arthroplasty on Endothelial Damage Marker Levels: A Prospective Comparative Study
Source: PLoS One. 2015 May 8;10(5):e0126663. doi: 10.1371/journal.pone.0126663 (PMC4425488; doi:10.1371/journal.pone.0126663)
Supplement: S1 IRB — (PDF) [file pone.0126663.s001.pdf]

長庚醫療財團法人人體試驗倫理委員會
臨床試驗變更案同意證明書

地　　址：333 桃園縣龜山鄉舊路村頂湖路123
傳　　真：03-3494549
聯絡人及電話：邱春櫻 03-3196200 ext. 3714
電子郵件信箱：cci@cgmh.org.tw

試驗名稱：電腦導航人工膝關節置換與傳統人工膝關節置換之前瞻性臨床及細胞動素之追蹤與比較研究

計畫編號：100-0038A3

本院案號：101-0050C（原100-0038A3）

試驗期間：102年01月16日起至104年12月31日止

trial number: 101-0050C
(previous 100-0038A3)
trial period: 2013-01-16 to 2015/12/31

主持人：高雄運動醫學科郭繼陽學術組教授級主治醫師

共同主持人：徐山琳、林子平、蕭家傑、翁聆修、王清貞、王逢興、周文毅

執行機構：長庚醫院高雄

同意計畫書版本：Version II, 101-11-10

同意之受試者同意書版本：Version V, 101-12-29

通過日期：102年01月16日

期中報告繳交頻率：一年一次

※請於到期前二個月繳交期中報告以利本會進行審查※

長庚醫療財團法人
人體試驗倫理委員會謝燦堂主席

中　華　民　國　102　年　01　月　25　日

【主持人須知】

一、實施人體研究計畫前，應擬定研究計畫，經人體試驗倫理委員會審查通過，始得為之。另醫療法所稱之人體試驗案及應用人體生物資料庫檢體進行之案件，尚需經衛生署核准，方可進行。人體試驗倫理委員會或主管機關命令中止/終止試驗案件時，不得繼續執行。

二、試驗進行前，主持人應確實核對試驗計畫書、受試者同意書等之正確版本，以及人體試驗倫理委員會與衛生署核准之試驗進行期間；醫療法所稱之人體試驗案，應於本院人體試驗倫理委員會與衛生署皆已核准，方可進行；並以人體試驗倫理委員會核准之同意臨床試驗證明迄日為試驗截止日。

三、應完全熟悉試驗藥品/醫療材料、醫療技術在試驗計畫書、最新主持人手冊及其他由試驗委託者提供的相關資訊中描述的使用方法。

長庚醫療財團法人人體試驗倫理委員會
同意臨床試驗證明書

地　址：333 桃園縣龜山鄉舊路村頂湖路123號2
傳　真：03-3494549
聯絡人及電話：葉倩吟 03-3196200 ext. 3701
電子郵件信箱：erinyeh@adm.cgmh.org.tw

查「電腦導航人工膝關節置換與傳統人工膝關節置換之前瞻性臨床及細胞動素之追蹤與比較研究」臨床試驗計畫案（計畫書：Version I, 99-12-30；受試者同意書：Version III, 100-3-11；本院編號100-0038A3，通過會期：100年03月01日，另依本會決議本案期中報告繳交頻率為每一年一次，試驗期間為100年03月17日起至103年03月16日止），已於100年03月17日經本院人體試驗倫理委員會審查通過，同意由高雄運動醫學科郭繼陽醫師、高雄運動醫學科徐山琳醫師、高雄脊椎科林子平醫師、高雄運動醫學科翁聆修醫師、高雄運動醫學科王清貞醫師、高雄研究區王逢興副教授、高雄運動醫學科周文毅醫師、高雄運動醫學科黃濤醫師依所提計畫進行臨床試驗。特此證明。

The underlined text means that the trial number " 100-0038A3" was approved on 2011/03/01 with the approved period of 2011/03/17 to 2014/03/16.
The enrolled patients were since 2011/03/17 to 2011/12/31. The samples after 2011/12/31 were not analyzed due to absence of funding (the ELISA analysis in the manuscript was performed by the salary of corresponding author. The protocol was extended to 2015/12/31 with the protocol number of " 101-0050C ".

長庚醫療財團法人
人體試驗倫理委員會謝燦堂主席

長庚醫療財團法人 研究計劃專用章

中　華　民　國　100　年　03　月　18　日

【主持人須知】

※自98年1月1日核准之新案，依規定繳交期中及結案報告，未繳者不得再申請新案。

一、應完全熟悉試驗藥品／醫療材料或醫療技術在試驗計畫書、最新主持人手冊及其他由試驗委託者提供的相關資訊中描述的使用方法。

二、應明瞭並遵守『藥品優良臨床試驗準則』和衛生署相關法規要求，善盡保護受試者之責任，並配合相關主管機關的查核。

三、應確保所有協助人體試驗的相關人員對試驗計畫書及試驗藥品／醫療材料或醫療技術有充分的了解，以及他們在人體試驗中相關的責任和工作。

四、試驗主持人應負責所有人體試驗相關的醫療決定。

長庚紀念醫院人體試驗倫理委員會
同意臨床試驗證明書

查「電腦導航系統人工膝關節置換手術之回溯性及未來追蹤研究」臨床試驗計畫案（本院案號96-1408B，試驗期間為97年2月20日起至99年12月31日止），已於97年2月20日經本院人體試驗倫理委員會審查通過，同意由高雄骨科郭繼陽醫師、黃濤醫師依所提計畫進行臨床試驗。特此證明。

財團法人長庚紀念醫院
人體試驗倫理委員會謝燦堂主席

中　華　民　國　97　年　2　月　21　日

【主持人須知】

一、　試驗執行前應協助試驗執行院區完成合約簽署並提供本會影本乙份。

二、　應完全熟悉試驗藥品／醫療材料或醫療技術在試驗計畫書、最新主持人手冊及其他由試驗委託者提供的相關資訊中描述的使用方法。

三、　應明瞭並遵守『藥品優良臨床試驗準則』和衛生署相關法規要求，善盡保護受試者之責任，並配合相關主管機關的查核。

四、　應確保所有協助人體試驗的相關人員對試驗計畫書及試驗藥品／醫療材料或醫療技術有充分的了解，以及他們在人體試驗中相關的責任和工作。

五、　試驗主持人應負責所有人體試驗相關的醫療決定。

六、　在受試者參加試驗與後續追蹤期間，試驗主持人應確保對受試者任何與試驗相關的不良反應，包括重要實驗室檢查值等，提供充分的醫療照護。當試驗主持人察覺試驗期間受試者有疾病需要醫療照護時，必須告知受試者。

七、　主持人須遵照『嚴重藥物不良反應通報辦法』及『藥品優良臨床試驗準則』之規定，於時效內完成通報。

八、　試驗主持人應依本院『人體試驗作業管理準則』規定繳交期中報告及結案報告，案件審議如有必要時須配合於會議列席報告。

九、　執行院區應與呈准之計劃書一致，有可能之收案地點應在原計劃書中載明；試驗執行院區若有任何修改或新增，則應送修正案審查，於接獲書面核准函後始可執行。

十、　其餘未竟事宜，請參照本院『人體試驗作業管理準則』辦理。
