# Supplementary material for: Effects of Computer Navigation versus Conventional Total Knee Arthroplasty on Endothelial Damage Marker Levels: A Prospective Comparative Study
Source: PLoS One. 2015 May 8;10(5):e0126663. doi: 10.1371/journal.pone.0126663 (PMC4425488; doi:10.1371/journal.pone.0126663)
Supplement: S1_Protocol — (DOC) [file pone.0126663.s002.doc]

**電腦導航人工膝關節置換與傳統人工膝關節置換之前瞻性臨床及細胞動素之追蹤與比較研究**

人工膝關節置換術( TKA)是目前最成功之骨科手術之一.但傳統人工膝關節置換除了角度無法完善精準之外，因術中對股骨骨髓腔之破壞(須做femoral intramedullary rodding以作為手術之依據)，可能會有肺栓塞、腦中風，甚至心肌梗塞之虞. 電腦導航人工膝關節置換術提供精確的人工關節置入角度及量化的膝部動態，理論上會比傳統人工膝關節置換有更好的預後及一貫性的結果.另外電腦導航之應用,可避免髓內器具對骨髓之侵犯及軟組織之破壞, 有微創手術之真實意義.可以減少手術血流動力學之不穩定以及減少各系統之併發症, 包括腦部,心血管系統, 肺部系統之栓塞以及腸胃系統不適、感染以及骨折之可能.

**預期成果：** 我們預期由相關之臨床結果與細胞動素研究，可以佐證電腦導航人工膝關節置換與傳統人工膝關節置換之差異，此差異可能減低手術併發症之發生及改善手術之短、中、長期結果。

**研究計畫之背景及目的**

**Background:**

Total knee arthroplasty (TKA) is one of the most successful surgery in orthopaedic field. However, occasional outliner from perfect alignment after prosthesis implantation and unavoidable complications are still concerns. The usage of navigation-assisted system on total knee arthroplasty has provided better accuracy of the component alignment and quantity in knee kinematics, which theoretically affords better and consistent functional outcome.

In addition to better alignment, the design of navigation system avoids violation of the medullary canal. This less invasive environment might be contributable to the less blood loss in the drainage bottle, less blood transfusion more stable hemodynamic status, fewer hospitalization days, and fewer complications.

Systemic emboli phenomena during preparation of the femur and tibia are well recognized during total knee arthroplasty. They are widely believed to be the cause of intraoperative hypotension and reduced cardiac output, which may lead to circulatory collapse, change of mental status or cerebral infarction. Kalairajah et al reported that navigation-assisted total knee arthroplasty, when compared with conventional jig-based surgery, significantly reduces systemic emboli as detected by transcranial Doppler ultrasonography. Church et al undertook a prospective, double-blind, randomized study to compare the cardiac emboli load by tranesophageal echocardiography and demonstrated that computer-assisted TKA resulted in the release of significant fewer systemic emboli than the conventional procedure using intramedullary alignment. The increased blood loss in conventional TKA may be due to intramedullary jigging of both femur and tibia, bleeding from sinusoids at the cut cancellous bone surfaces with continuous suction drainage and more soft tissue dissection during balancing of the prosthesis. We have underwent more than 730 computer-assisted TKA since 2005. Our data shows less bleeding and fewer transfusion after navigation assisted TKA. We wish to further follow-up and delineate the differential clinical outcomes and perioperative cytokines between computer-assisted TKA and conventional TKA.

The specific aims of this project are as follows:

1. Prospective follow-up and comparative analysis of early, midterm, and long-term surgical results and complications between navigation assisted TKA and conventional TKA.

2. Prospective comparative analysis of cytokines from blood, tissues, and drainage between navigation assisted TKA and conventional TKA.

3. Prospective follow-up of surgical results of navigation-assisted TKA.

**Anticipated results:**

We anticipate decreased complication rate and improved functional result of total knee arthroplasty with the assistance of navigation system. We also anticipate that the differential expression of cytokines may indicate fewer tissue damage and better long-term functional results in computer-assisted TKA.

**[目的、重要性]**

隨著人口老化問題, 膝部人工關節置換術之需求日益增加, 對於人工關節置換術成功標準的要求亦隨著醫學科技進步而愈加嚴格,另外因接受人工膝關節置換之病患大都為老年人，手術之安全性及合併症之預防亦是重要的課題。電腦導航系統的發展也是順應人工膝關節置換手術的高嚴謹度及精緻度而出現。

本實驗希望探討電腦導航系統應用於全人工膝關節置換手術之相關研究，了解電腦導航系統應用於全人工膝關節置換手術之優缺點，改善因為人工膝關節置換術所引起的相關併發症及得到一個更優良的手術結果，也希望本研究對需要接受膝部人工膝關節置換的骨科病患有實質的幫忙。

**[國內外有關本計劃之研究情況]**

國外這兩年有將近百篇論文發表使用電腦導航系統於全人工膝關節置換手術之相關研究如Chauhan,在2004年 *J Bone Joint Surg Br,* 86(6): 818-23 發表的Computer-assisted total knee replacement. A controlled cadaver study using a multi-parameter quantitative CT assessment of alignment (the Perth CT Protocol)中提出電腦導航系統於全人工膝關節置換手術可提高人工膝關節置入之精確性.Church在2007年*J Bone Joint Surg Br,* 89(4): 481-5發表Embolic phenomena during computer-assisted and conventional total knee replacement 中提出使用電腦導航系統於全人工膝關節置換手術可減低手術引起栓塞之併發症.

國內方面, 嘉義長庚許文蔚榮譽院長執navigation TKA之牛耳，且多次於國外重要會議發表演講，成功大學楊俊佑教授在台灣醫誌發表電腦導航系統於全人工膝關節置換手術的經驗,而本醫療團隊成員周文毅醫師使用電腦導航系統於全人工膝關節置換手術的報告發表於Journal of Arthroplasty。本科系近年對電腦導航系統於全人工膝關節置換手術有不錯的成果，多次於國內外相關會議發表, 並寫成paper投稿中，藉由這些experiences及我們對電腦導航全人工膝關節置換手術之投入，我們相信可對全人工膝關節置換手術之改進作出貢獻。

[References]

## 1. Bauwens, K.; Matthes, G.; Wich, M.; Gebhard, F.; Hanson, B.; Ekkernkamp, A.; and Stengel, D.: Navigated total knee replacement. A meta-analysis. *J Bone Joint Surg Am,* 89(2): 261-9, 2007.

2. Chang C. W.; Yang C. Y: Kinematic navigation in total knee replacement - experience from the first 50 cases. J Formos Med Assoc 105(6):468-74,2006.

## 3. Chauhan, S. K.; Clark, G. W.; Lloyd, S.; Scott, R. G.; Breidahl, W.; and Sikorski, J. M.: Computer-assisted total knee replacement. A controlled cadaver study using a multi-parameter quantitative CT assessment of alignment (the Perth CT Protocol). *J Bone Joint Surg Br,* 86(6): 818-23, 2004.

## 4. Church, J. S.; Scadden, J. E.; Gupta, R. R.; Cokis, C.; Williams, K. A.; and Janes, G. C.: Embolic phenomena during computer-assisted and conventional total knee replacement. *J Bone Joint Surg Br,* 89(4): 481-5, 2007.

## 5. Eum, D. S.; Lee, H. K.; Hwang, S. Y.; and Park, J. U.: Blood loss after navigation-assisted minimally invasive total knee arthroplasty. *Orthopedics,* 29(10 Suppl): S152-4, 2006.

## 6. Fulkerson, E.; Valle, C. J.; Wise, B.; Walsh, M.; Preston, C.; and Di Cesare, P. E.: Antibiotic susceptibility of bacteria infecting total joint arthroplasty sites. *J Bone Joint Surg Am,* 88(6): 1231-7, 2006.

## 7. Han, H. S.; Seong, S. C.; Lee, S.; and Lee, M. C.: Rotational alignment of femoral components in total knee arthroplasty: nonimage-based navigation system versus conventional technique. *Orthopedics,* 29(10 Suppl): S148-51, 2006.

## 8. Kalairajah, Y.; Cossey, A. J.; Verrall, G. M.; Ludbrook, G.; and Spriggins, A. J.: Are systemic emboli reduced in computer-assisted knee surgery?: A prospective, randomised, clinical trial. *J Bone Joint Surg Br,* 88(2): 198-202, 2006.

## 9. Kalairajah, Y.; Simpson, D.; Cossey, A. J.; Verrall, G. M.; and Spriggins, A. J.: Blood loss after total knee replacement: effects of computer-assisted surgery. *J Bone Joint Surg Br,* 87(11): 1480-2, 2005.

## 10. Kim, Y. H.; Kim, J. S.; and Yoon, S. H.: Alignment and orientation of the components in total knee replacement with and without navigation support: a prospective, randomised study. *J Bone Joint Surg Br,* 89(4): 471-6, 2007.

## 11. Martin, A.; Wohlgenannt, O.; Prenn, M.; Oelsch, C.; and Strempel, A.: Imageless Navigation for TKA Increases Implantation Accuracy. *Clin Orthop Relat Res,* 460: 178-84, 2007.

## 12. Middleton, F. R., and Palmer, S. H.: How accurate is Whiteside's line as a reference axis in total knee arthroplasty? *Knee,* 14(3): 204-7, 2007.

## 13. Mombert, M.; Van Den Daelen, L.; Gunst, P.; and Missinne, L.: Navigated total knee arthroplasty: a radiological analysis of 42 randomised cases. *Acta Orthop Belg,* 73(1): 49-54, 2007.

## 14. Mullaji, A.; Kanna, R.; Marawar, S.; Kohli, A.; and Sharma, A.: Comparison of limb and component alignment using computer-assisted navigation versus image intensifier-guided conventional total knee arthroplasty: a prospective, randomized, single-surgeon study of 467 knees. *J Arthroplasty,* 22(7): 953-9, 2007.

## 15. Parvizi, J.; Mui, A.; Purtill, J. J.; Sharkey, P. F.; Hozack, W. J.; and Rothman, R. H.: Total joint arthroplasty: When do fatal or near-fatal complications occur? *J Bone Joint Surg Am,* 89(1): 27-32, 2007.

## 16. Patai, J.; Janositz, G.; Mecs, L.; and Toth, K.: Navigated total knee arthroplasty in a patient with severe diaphyseal deformities. *Acta Orthop Belg,* 73(4): 536-40, 2007.

## 17. Perillo-Marcone, A., and Taylor, M.: Effect of Varus/Valgus Malalignment on Bone Strains in the Proximal Tibia After TKR: An Explicit Finite Element Study. *J Biomech Eng,* 129(1): 1-11, 2007.

## 18. Picard, F.; Deakin, A. H.; Clarke, J. V.; Dillon, J. M.; and Gregori, A.: Using navigation intraoperative measurements narrows range of outcomes in TKA. *Clin Orthop Relat Res,* 463: 50-7, 2007.

## 19. Picard, F.; Gregori, A.; Dean, F.; Mennessier, A.; and Dillon, J.: Computer-assisted dynamic total knee arthroplasty using Whiteside's line for alignment. *Orthopedics,* 29(10 Suppl): S104-7, 2006.

## 20. Rauh, M. A.; Boyle, J.; Mihalko, W. M.; Phillips, M. J.; Bayers-Thering, M.; and Krackow, K. A.: Reliability of measuring long-standing lower extremity radiographs. *Orthopedics,* 30(4): 299-303, 2007.

## 21. Seon, J. K., and Song, E. K.: Navigation-assisted less invasive total knee arthroplasty compared with conventional total knee arthroplasty: a randomized prospective trial. *J Arthroplasty,* 21(6): 777-82, 2006.

## 22. Spencer, J. M.; Chauhan, S. K.; Sloan, K.; Taylor, A.; and Beaver, R. J.: Computer navigation versus conventional total knee replacement: no difference in functional results at two years. *J Bone Joint Surg Br,* 89(4): 477-80, 2007.

## 23. Spirt, A. A.; Assal, M.; and Hansen, S. T., Jr.: Complications and failure after total ankle arthroplasty. *J Bone Joint Surg Am,* 86-A(6): 1172-8, 2004.

## 24. Stockl, B.; Nogler, M.; Rosiek, R.; Fischer, M.; Krismer, M.; and Kessler, O.: Navigation improves accuracy of rotational alignment in total knee arthroplasty. *Clin Orthop Relat Res,* (426): 180-6, 2004.

## 25. Taggart, T.; Kerry, R. M.; Norman, P.; and Stockley, I.: The use of vancomycin-impregnated cement beads in the management of infection of prosthetic joints. *J Bone Joint Surg Br,* 84(1): 70-2, 2002.

26. Weng, Y. J; Hsu R.W.W; Hsu W.H: Comparison of computer-assisted navigation and conventional insttumentation for bilateral total knee arthroplasty. J of Arthroplasty 24(5):668-73, 2009.

## 27. Tingart, M.; Luring, C.; Bathis, H.; Beckmann, J.; Grifka, J.; and Perlick, L.: Computer-assisted total knee arthroplasty versus the conventional technique: how precise is navigation in clinical routine? *Knee Surg Sports Traumatol Arthrosc*, 2007.

## 28. Werner, F. W.; Ayers, D. C.; Maletsky, L. P.; and Rullkoetter, P. J.: The effect of valgus/varus malalignment on load distribution in total knee replacements. *J Biomech,* 38(2): 349-55, 2005.

**27.**  Chou,Y.I. ; Ko,J.Y.; Wang, C.J.; Wang, F.S.; Wu,R.W.; Wong, T. :

Navigation-Assisted Total Knee Arthroplasty for a Knee With Malunion of the Distal Femur- Case report . *J Arthroplasty,* 23(8): 1239e13-17, 2008.

**研究方法及進行步驟：**

**Research design of the initial 3 years**

We shall prospectively follow-up and compare the baseline data; imaging, cytokines, and surgical results between conventional total knee arthroplasty and navigation-assisted total knee arthroplasty performed between 2011 and 2015. The data include preoperative parameter; perioperative condition differential expression of cytokines and postoperation function follow up.

**Materials and methods**

Between January 2011 and December 2015, at least 30 consecutive patients undergoing a primary navigation-assisted TKA for osteoarthritis, rheumatoid arthritis or other disorders will be considered for inclusion in the study. Another 30 or more consecutive conventional primary TKA will also be included as the comparative group. Before surgery, patients’ age, height, weight basic data, and body mass index (BMI) will be recorded. Their pre-operative anesthetical state will be defined according to the American Society of Anaesthesiology classification (ASA grade).

Indications for TKA include advanced osteoarthritis, rheumatoid arthritis and traumatic arthrosis. Contraindications include knee sepsis with previous osteotomylitis, a remote source of ongoing infection, server cardio-vascular disease precluding surgical procedure, and the presence of a well-functioning knee arthrodesis. The use of acetylsalicylic acid will be stopped 1 week before the surgery and continued on the next day after the surgery. Other nonsteroidal anti-inflammatory drugs are not restricted before or after surgery. Before surgery, 10cc peripheral blood will be obtained from all patients as a baseline. Conventional TKA will be performed in a bloodless field using a pneumatic tourniquet at a pressure of 300mm Hg after a single injection of antibiotic (cefazolin sodium 1g). A midvastus approach is used through a midline skin incision of 10-12cm. The femur and / or tibia bone cuts are adjusted via an intramedullary guide. The surgical procedure is performed according to the manufactor’s instruction. The prosthesis is implanted with cement fixation.

A navigation-assisted TKA is performed on the patients with a similar midvastus approach through a midline skin incision. The symposium is partially removed to enable precise registration. A CT-free navigation System (Vector Vision; Brain LAB, Heimstetten, Germany) with anatomical mapping of the knee and kinematics analysis is employed to generate a working model of the patient’s knee. An infrared camera is equipped to track two fixed references with marker spheres that are fixed to the distal femur (4mm pins) and the proximal tibia (3mm pins) using two bi-cortical half-pins. The center of the femoral head, ankle landmarks and mapping of the distal femur and proximal tibia are registered. The femoral component is referenced parallel to the anterior cortex of the distal femur. A multiple referencing method using the epicondylar line, Whiteside line and posterior condylar line is adapted for the rotation of the femoral component. The rotation of the tibial component is adjusted to that of the femoral component and is parallel to the axis between the medial third of the tibial tuberosity and the center of the tibial plateau. An additional extramedullary guiding rod is used as a reference of the midpoint of the anterior ankle joint to assist with the determination of the rotation of tibial component. An accurate bone cut is achieved without intramedullary insult and the prosthesis (LPS-Flex system, Nexgen; Zimmer, Warsaw, IN, USA) is implanted with cement fixation. The ideal mechanical axis of within a 1.0° deviation is obtained after soft tissue balancing with the aid of a real-time computer screen. The tourniquet is released when the wound is closed. A 1/8 inch haemovac is inserted as a closed drainage system. Another 10cc peripheral blood will be obtained the next day (about 24 hours) 3 days and 2 weeks after surgery. Hemovac drainage will be obtained about 24 hours after TKA.

Post-operative data analyze

The hemodynamic status and early complications will be evaluated. The grade of the American Society of anesthesiology, tourniquet time, amount of blood transfusion, blood drainage from the wound, and day of hospitalization will be recorded. The expressions of cytokines related to pain, tissue damage, inflammation, and bone metabolism will be measured.

Standardized radiographs and long-leg standing radiographs will be obtained six weeks after surgery and at follow-ups to determine the weight-bearing femoro-tibial components angle and the mechanical axis.

**Statistical analysis**

All values are expressed as mean ± standard error. Data will be analysed using SPSS 15 software (SPSS, Chicago, IL, USA). Descriptive statistics will be obtained using X^2^ distributions and one-factor analsysis of variance.

**Research design for the short-term, mid-term and long-term follow up**

Two, 5 and 10 years surgical follow-ups will be performed on patients undergoing conventional or navigation-assisted TKA between 2011 and 2015. Complications and function outcomes is analyzed. PE wear, implant loosening, infection rate and activity function scores are evaluated. Knee society function score and / or Tegner activity scale are used.

**預期完成之工作項目及具體成果：**

**預期試驗效果**

1. 第一年至第三年我們預期可比較傳統人工膝關節置換術及電腦導航人工膝關節置換有關末稍血及引流液之相關細胞動素之數據，得到有統計意義的結果，另外短期性併發症之研究可以作初步探討。

2. 第二年、第五年及第十年作短期、中期及長期之手術結果追蹤，我們亦預期電腦導航人工膝關節置換會有較好的結果。

**可能傷害及處理**

1. 電腦導航人工膝關節置換術與傳統全人工膝關節置換手術之副作用及危險無明顯不同，依文獻電腦導航人工膝關節置換可能更低。
2. 需於股骨及脛骨作定位器之固定, 可能引起骨折或感染，但本院使用之導航儀器(Brain Lab)並無類似報告(有明顯外傷或意外除外，如果明顯外傷，傳統人工膝關節可能有更嚴重之關節周圍骨折)。

3.緊急狀況之處理

（如有術後併發症之發生, 例如骨折或感染請聯絡骨科系郭繼陽教授，電話：07-7317123-8003。

**對於學術研究、國家發展之貢獻**

人工膝關節置換術於世界骨科學領域已接近瓶頸階段, 電腦導航微創手術觀念之引入在人工膝關節置換術帶入新的發展契機,加入電腦導航系統應用於全人工膝關節置換手術之相關研究對骨科人工膝關節置換術的改進會有顯著幫忙, 另外於電腦導航系統人工膝關節置換手術研究的成就, 可能會提高發展國際醫療的水準, 使台灣的人工膝關節置換術在國際聲譽上提高。
